# Cytotoxic and Apoptogenic Sesquiterpenoids from the Petroleum Ether Extract of *Artemisia aucheri* Aerial Parts

**Published:** 2019

**Authors:** Leila Hosseinzadeh, Yalda Shokoohinia, Mehri Arab, Elnaz Allahyari, Mahdi Mojarrab

**Affiliations:** a *Pharmaceutical Sciences Research Center, School of Pharmacy, Kermanshah University of Medical Sciences, Kermanshah, Iran. *; b *Student Research Committee, Kermanshah University of Medical Sciences, Kermanshah, Iran.*

**Keywords:** Artemisia aucheri, Sesquiterpenoid, Cytotoxicity, Apoptosis, Davanone derivatives

## Abstract

Different types of *Artemisia aucheri* extracts were reported to have various biological activities including a cytotoxic effect on some cancer cell lines. We investigated the antiproliferative activity of isolated sesquiterpenoids from petroleum ether extract of *Artemisia aucheri* (*A. aucheri*) aerial parts on SK-N-MC, MCF-7, and A2780 cell lines. Phytochemicals from the petroleum ether cold macerated extract were isolated using normal phase vacuum liquid chromatography and high pressure liquid chromatography (VLC and HPLC) and the structures of the components were determined by spectroscopic means. Cell viability was determined by 3-(4,5- dimethylthiazol-2yl)-2,5-diphenyltetrazolium bromide assay. Activation of caspases-3 and -9 was evaluated using a spectrophotometer. Mitochondrial membrane potential (MMP) was measured using rhodamine 123 fluorescent dye. Two tetrahydrofuran- type sesquiterpenoids, hydroperoxide of davanone (1) and hydroxydavanone (2) were isolated and characterized. Between these compounds, compound 1 exhibited more potent activity against the MCF-7, SK-N-MC and A2780 cell lines with IC_50_ values of 8.45 ± 0.81 µg/mL, 9.60 ± 1.32 µg/mL and 10.9 ± 2.03 µg/mL in A2780, MCF-7 and SK-N-MC cells, respectively. Compound 1 inhibited the growth of human cancer cells by induction of apoptosis. To the best of our knowledge, this is the first comprehensive study on cytotoxic and apoptotic mechanism of two davanone derivatives isolated from *A. aucheri* in human cancer cells. Overall, our data suggest that hydroperoxide of davanone (1) should be further studied *in-vivo *as a potential antitumor agent.

## Introduction

The potential usage of cytotoxic agents from natural origin in cancer treatment has resulted in an increased focus on introduction of bioactive plant- derived products to medicine (1). *Artemisia aucheri* Boiss. (Asteraceae) with Persian name of “Dermaneye koohi” is regarded as a wildly growing species in Iran (2). Analysis of volatiles from the aerial parts of the species has revealed the presence of some oxygenated monoterpenoids as major constituents (3-9) which is in conformity with the reported secondary metabolites from *A. aucheri* in a phytochemical study (10). *In-vitro *antifungal (5, 11 and 12), antimicrobial (13) and acaricidal (14) activities of the volatile oil and crude extract of the species have also been reported. Extracts of *A. aucheri *have exhibited *in-vitro *and *in-vivo *anti-leishmanial activity (15-17). Antimalarial potential of dichloromethane extract of the species in a cell free β-hematin formation assay (18) and *in-vivo* activity of ethanolic extract on chloroquine- sensitive strain of *Plasmodium* *berghei* (19) have been reported. Various extracts (20) and different fractions of petroleum ether extract (21) showed potent cytotoxic properties.

Hydroalcoholic extract of the species has the ability to accelerate the skin wound healing process (22) and exert protective activity against thioacetamide-induced hepatotoxicity (23). Antioxidant effect of methanolic (24) and aqueous (25) extracts as well as hypocholesterolemic and antiatherosclerotic effects of ethanolic extract (26, 27) are of other reported activities.

 In continuation of our previous studies on cytotoxicity of *A. aucheri* (20, 21), further phytochemical study on petroleum ether extract was carried out and cytotoxic effects of isolated secondary metabolites were evaluated on human cancer cell lines.

## Experimental

*General experimental procedures*

The preparation of the fractions of petroleum ether extract was carried out with silica gel 60 (Merck, 0.040-0.063 mm). Silica gel 60 F-254 (Merck, Aluminum sheet) was used for TLC analyses. Spots were detected by spraying cerium sulfate/molybdate reagent followed by heating (110 °C for 5 min). The chromatographic system for semi preparative HPLC consisted of a YL9111S binary pump, a YL9160 PDA detector and a Eurospher II 100-10 Si (250 × 20 mm ID., 10 μm) column (Knauer, Germany). NMR spectra were recorded on a Bruker Avance 500 MHz spectrometer and chemical shifts are given in δ (ppm). Mass analyses were performed with an Agilent 6410 Series mass spectrometer equipped with an electrospray ionization source. 

*Reagents and chemicals*

All solvents used for extraction (petroleum ether 40-60), fractionation and further purification (n- heptane, n- hexane and ethyl acetate) were purchased from Caledon (Canada) and Scharlau (Spain). 3-(4,5- dimethylthiazol-2yl)-2,5 – diphenyltetrazolium bromide (MTT), rhodamine 123, and caspases activity detection kit were procured from Sigma Aldrich (USA). Cell culture medium, penicillin–streptomycin solution, and fetal bovine serum (FBS) were purchased from Gibco (USA). Biorad protein assay kit was obtained from Sigma (USA). All tissue culture wells were from Becton Dickinson (USA).

*Cell culture conditions*

MCF-7 (Human breast carcinoma), SK-N-MC (Human neuroblastoma), and A2780 (human ovarian carcinoma) cell lines were obtained from Pasteur Institute (Tehran, Iran) and maintained at 37 ºC in a humidified atmosphere (90%) containing 5% CO_2_. The cells were cultured in Dulbecco’s modified Eagle’s medium (DMEM-F12) with 10% (v/v) heat-inactivated fetal bovine serum, 100 UmL^−1^ penicillin and 100 mgmL^−1^ streptomycin.

*Plant material*

Aerial parts of *A. aucheri *were collected from Chahar-Bagh region, Golestan province, Iran, in September 2011. The plant was identified by Mr. S. A. Hosseini (Agricultural and Natural Resources Research Center of Golestan Province), Gorgan, Iran. A morphological comparison between collected sample and deposited voucher specimen (No. 2383) was accomplished.

*Extraction and isolation*

The air dried aerial parts of *A. aucheri* (330 g) were exhaustively extracted with Petroleum ether (40-60) at room temperature. After removal of solvent *in-vacuo*, the residue (3.51 g) was partitioned using vacuum liquid chromatography (VLC) with increasing polarity of solvent from n- heptane to ethyl acetate (EtoAc). Aliquots with similar TLC profile were combined to give 9 fractions. 

The most potent cytotoxic fractions (21) were subjected to further phytochemical analyses. Fraction 5 was purified by preparative HPLC using normal phase solvents (n- Hexane: ethyl acetate, 50:50). Eight subfractions were achieved as 4A to 4H in which 4D and 4F were pure sesquiterpenoids, compound 1 (10.6 mg) and compound 2 (12.1 mg), respectively. Compound 2 was obtained from other fractions (6, 7 and 8) as well to get 15.3 mg totally.

*Cytotoxicity assay*

Cytotoxic effects of the isolated compounds were studied in MCF-7, SK-N-MC and A2780 cell lines by MTT assay. Exponentially growing mammalian cells (on 96-well plates) were exposed for 24 h to different concentrations (0-100 µg/mL) of compounds 1 and 2. The control cells were kept in medium containing 0.1% DMSO. After 24 h of incubation, the medium was removed and 0.1 mg/well of MTT were added to the cells, and the plates were further incubated for 3 h at 37 °C. The formazan crystals were solubilized in 0.1 mL of dimethyl sulfoxide and the optical density (OD570) was measured using a microplate reader (BioTek Instruments, USA). IC_50_ values were calculated by plotting log10 of the percentage of proliferation versus drug concentration. 

*Measurement of mitochondrial membrane potential*

Mitochondrial membrane potential (MMP) was evaluated by rhodamine 123 fluorescent dye. Depolarization of MMP during cell apoptosis results in the loss of rhodamine 123 from the mitochondria and a decrease in intracellular fluorescence intensity (21, 28). After that, treatment cells were incubated with rhodamine 123 for 30 min at 37 °C. The fluorescence was measured at an excitation wavelength of 488 nm and an emission wavelength of 520 nm using a fluorescence microplate reader (BioTek, H1M, USA).

*Determination of Caspase-3 and -9 activities*

The caspase-3 and -9 activities were measured by commercial caspase assay kit (Sigma, USA), according to the manufacturer’s protocol. The kit is based on the hydrolysis of the peptide substrate Ac-DEVD-pNA (for caspase-3), and Ac-LEHD-pNA (for caspase-9) resulting in the release of the *p*-nitroaniline (pNA) moiety. Briefly, 1 × 10^6^ cells were collected and lysed with 50 μL of chilled lysis buffer and incubated on ice for 10 min. The cell lysates were centrifuged at maximum speed for 5 min at 4 °C. Then 10 μL of cell lysate was combined with an equal amount of substrate reaction buffer containing a caspase-3 and 9 colorimetric substrates. After 2 h of incubation at 37 °C, the pNA light emission was quantified using a microplate reader at 405 nm (BioTek, H1M.). Comparison of the absorbance of pNA from an apoptotic sample with an un-induced control allowed determination of the fold increase in caspase-3 and 9 activities. The protein content was determined by the Bradford method using the bovine serum albumin as a standard.

## Results

*Isolation and structure elucidation of compounds 1 and 2*

**Figure 1:**
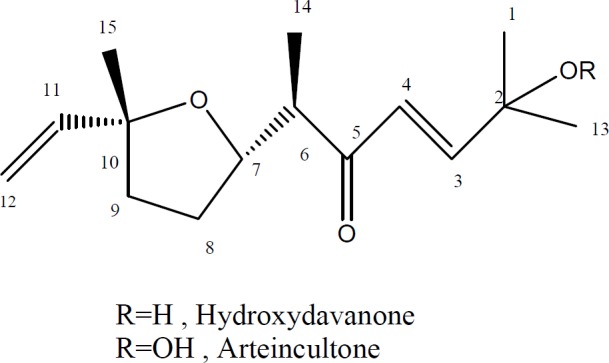
Chemical structures of compounds 1 and 2

From Petroleum ether extract of *A. aucheri* two terpenoids including hydroperoxide and hydroxyl derivatives of davanone were isolated. Isolated compounds (Figure 1) were identified by comparison of their NMR and MS spectral data to those reported in the literature (29, 30). ^1^H-NMR and mass spectral data of the isolated compounds are as follows:

*Hydroperoxide of davanone (arteincultone) (1)*

^1^H-NMR (500 MHz, CDCl3): δ 8.02 (OOH, br s), 6.86 (1H, d, J = 16.1 Hz, H-3), 6.40 (1H, d, J = 16.1 Hz, H-4), 5.90 (1H, dd, J = 17.2,10.7 Hz, H-11), 5.18 (1H, dd, J = 17.2, 1.5 Hz, H-12a), 4.99 (1H, dd, J = 10.7, 1.5 Hz, H-12b), 4.16 (1H, ddd, J = 8.3, 8.3, 6.0 Hz, H-7), 2.96 (1H, dq, J = 8.3, 7.0 Hz, H-6), 2.03 (1H, m, H-8a), 1.88 (1H, m, H-9a), 1.76 (1H, m, H-9b), 1.68 (1H, m, H-8b),1.39 (3H, s, H-1), 1.39 (3H, s, H-13), 1.25 (3H, s, H-15), 1.05 (3H, d, J = 7.0 Hz, H-14). ESIMS: *m/z *269 [M + H]^+^, 291 [M+ Na]^+^

*Hydroxydavanone (2)*

^1^H-NMR (500 MHz, CDCl3): δ 6.92 (1H, d, J = 15.8 Hz, H-3), 6.42 (1H, d, J = 15.8 Hz, H-4), 5.90 (1H, dd, J = 17.2, 10.7 Hz, H-11), 5.18 (1H, dd, J = 17.2, 1.6 Hz, H-12a), 4.97 (1H, dd, J = 10.7, 1.6 Hz, H-12b), 4.22 (1H, ddd , J = 8.3, 8.3, 6.0 Hz, H-7), 2.94 (1H, dq, J = 8.0, 7.0 Hz, H-6), 2.01 (1H, m, H-8a), 1.89 (1H, m, H-9a), 1.75 (1H, m, H-9b), 1.66 (1H, m, H-8b), 1.38 (3H, s , H-1), 1.38 (3H, s , H-13), 1.26 (3H, s , H-15), 1.04 (3H, d, J = 7.0 Hz, H-14). ESIMS: *m/z *253 [M + H]^+^, 275 [M+ Na]^+ ^

**Figure 2:**
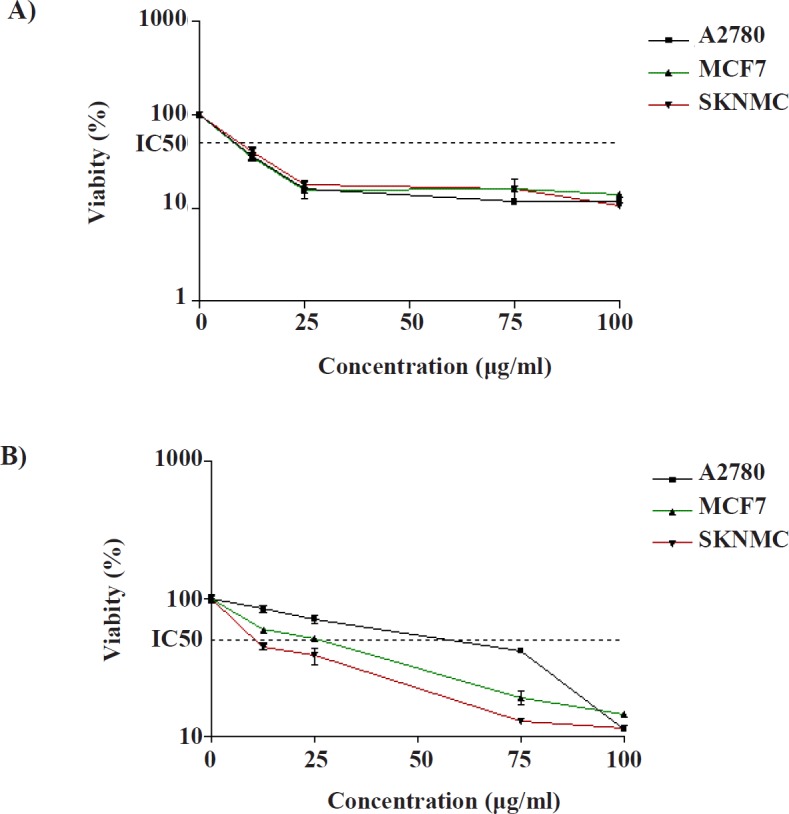
Cytotoxic effects of (A) compound 1 and (B) compound 2 in SK-N-MC, MCF-7 and A2780 cancer cells. The cells were incubated with different concentrations of compounds for 24 h. The cell proliferation inhibition was determined by MTT assay as described under materials and methods. Data are presented as mean ± SEM (N = 3)

**Figure 3:**
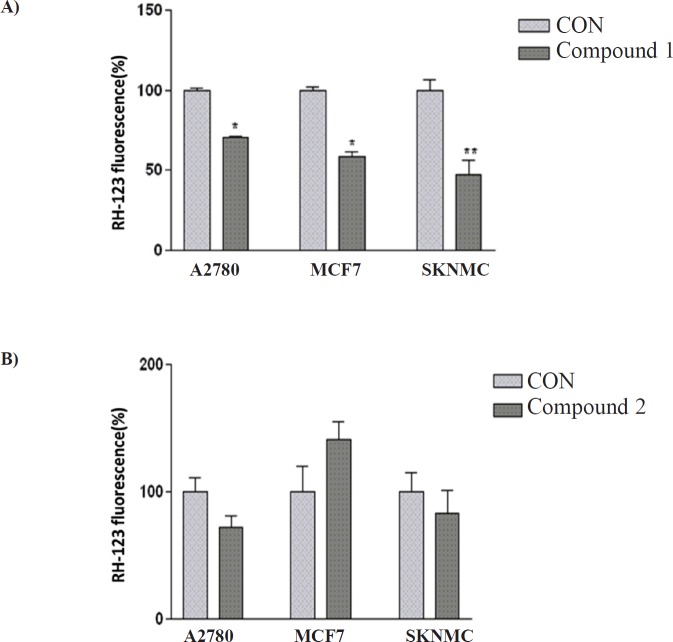
The effect of (A) compound 1 and (B) compound 2 on MMP in SK-N-MC, MCF-7 and A2780 cancer cells. Data are presented as mean ± SEM, *P < 0.05, **P < 0.01 *vs. *control

**Figure 4:**
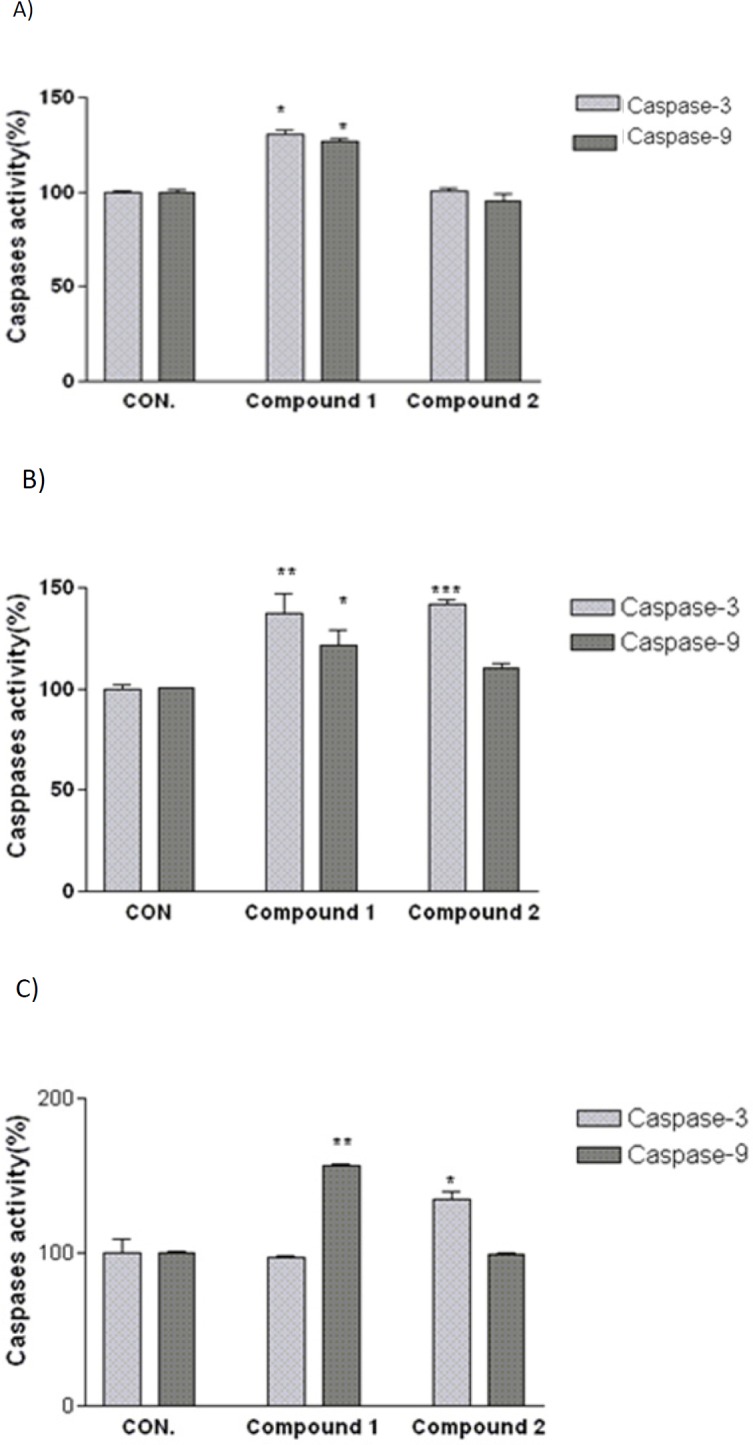
Involvement of activation of caspases in the induction of apoptosis on (A) SK-N-MC, (B) A2780, and (C) MCF-7 human cancer cells. Cells were incubated with IC_50 _concentration of the indicated compounds and harvested at 24 h and cell lysates were assayed using microplate reader for activation caspases. Significant differences were compared with the control. Data are presented as mean ± SEM. *P < 0.05, **P < 0.01 and ***P < 0.001 versus control

*Cytotoxic activities of the compounds*

To examine the *in-vitro* anti-cancer efficacy of the compounds, the potency of isolated compounds to induce cell death was determined on SK-N-MC, MCF-7, and A2780 cell lines under MTT method. As shown in Figures 2a and 2b, exposure to compound 1 for 24 h resulted in a concentration dependent decrease in cell viability, with approximate IC_50_ values of 8.45 ± 0.81 µg/mL, 9.60 ± 1.32 µg/mL and 10.9 ± 2.03 µg/mL in A2780, MCF-7, and SK-N-MC cells, respectively. These values are below 20 µg/mL, which indicate that compound 1 potentially presents an interesting cytotoxic activity towards three human carcinoma cell lines (31). Compound 2 can be accepted as potent cytotoxic agents against SK-N-MC because its IC_50_ value (IC_50 _= 10 ± 0.91 µg/mL) is below 20 μg/mL. Moreover, it showed moderate cytotoxic effect against MCF- 7 (IC_50 _= 25.4 ± 4.21 µg/mL) and A2780 (IC_50_ = 41.69 ± 2.87 µg/mL) cell lines. 

*Effect of isolated compounds on mitochondrial membrane potential (MMP)*

To characterize the changes in mitochondrial events induced by treatments with isolated compounds, the collapse of MMP in SK-N-MC, A2780 and MCF-7 cell lines were monitored with the rhodamine 123.

Decrease of mitochondrial membrane potential caused by the compound 1- induced damage of the outer membrane resulted in the loss of the dye from the mitochondria and a decrease in intracellular fluorescence, so that compound 1 signiﬁcantly decreased MMP in three human carcinoma cell lines. Moreover, the result indicated that compound 2 was not able to change MMP in any studied cell lines (Figure 3).

*Effect of isolated compounds on caspase 3 and 9 activities*

As shown in Figure 4, after 24 h treatment with IC_50_ concentration of compound 2, caspase-3 activity in A2780 and MCF-7 cells was significantly increased. To determine which apoptotic pathway is activated by this compound, we evaluated the activation of caspase 9, the apical protease in intrinsic pathways. Compound 2 was not able to increase the activity of caspase 9 in above mentioned cell lines. In addition, the obtained results indicated that compound 1 considerably increases caspase-3 and -9 enzyme activities in A2780 and SK-N-MC cell lines while no increase in caspase -3 activity was shown after exposure to compound 1 in MCF-7 cell line. Activity of caspase-9 was found to increase significantly after 24 h exposure to IC_50_ concentration of compound 1 in MCF-7 cell line.

## Discussion

In this study, two tetrahydrofuran- type sesquiterpenoids (hydroperoxide of davanone (1) and hydroxydavanone (2)) were isolated from petroleum ether extract of *A*. *aucheri.* We first reported a cell growth inhibitory effect for isolates in human carcinoma cell lines. Exposure to the isolated sesquiterpenoids for 24 h resulted in dose-dependent inhibition of carcinoma cell growth, as demonstrated by MTT assay. Between the isolates, compound 1 showed higher inhibitory potency against the growth of human carcinoma cell lines. When exposed to cytotoxic agents, the cells might suffer death or a stop in their cell cycle, which in turn can trigger death by apoptosis if cells cannot overcome the damage (32). Apoptosis is a common mechanism involved in the toxicity of many anticancer drugs, including those from natural origin, and is considered as desired mode of cell death in chemotherapy (33). Here, we investigated whether or not the cytotoxic effects of isolated compounds on carcinoma cell lines were mediated via apoptosis by examining well-characterized apoptosis markers. Activation of caspase cascade is critical in the initiation of apoptosis in various biological systems. A member of this family, caspase-3 has been identified as being a key mediator of apoptosis (34). Our results showed that 24 h treatment with IC_50_ concentration of compound 2 increased caspase-3 activation in A2780 and MCF-7 cell lines. Additionally, compounds 1 increased activity of caspase 3 in A2780 and SK-N-MC cell lines, significantly. Mitochondria are double membrane-bound organelles that play significant roles in activating apoptosis through intrinsic pathway in mammalian cell (35). During apoptosis, the mitochondrial membrane potential (MMP) decreases. A decrease in MMP leads to matrix condensation and the release of cytochrome c from the mitochondrial intermembrane space. The release of cytochrome c triggers the activation of the caspase 9 which goes on to activate the effector caspases (36). The results indicated that compound 1 was able to significantly decrease MMP and also increased caspase 9 activity in three human carcinoma cell lines. These evidences showed that compound 1 is capable of triggering apoptosis via the mitochondrial pathway. Compound 2 was not able to change caspase-9 activity and MMP in the MCF-7 and A2780 cell lines, significantly; indicating that the mitochondrial apoptotic pathway was not activated. Moreover, it appears that compound 2 induced cell death through necrosis in SK-N-MC cell line. 

Among different extracts of various species of the genus *Artemisia*, petroleum ether extracts of *A. ciniformis* and *A. aucheri* have been reported to be the most potent cytotoxic samples against human erythroleukemic (37), human neuroblastoma, breast adenocarcinoma and ovarian cancer cell lines (20), respectively. Moderate to potent cytotoxicity of isolated sesquiterpenoids from the genus *Artemisia* have been shown against different cell lines which human cervix adenocarcinoma (38), human lung cancer 95-D (39), human colon carcinoma and non–small cell lung cancer cell lines (40) are some of them. Previously, synthesis of hydroxydavanone (41) and isolation of the compound from *Artemisia* genus (29, 42-48) as well as studies on antifungal activity of hydroxydavanone (49) had been reported. A davanone hydroperoxide derivative named arteincultone has been isolated from *Artemisia* genus, as well (29, 46 and 49-50). To the best of our knowledge, there is no previous report on isolation of aforementioned compounds from *A*. *aucheri* and on cytotoxicity of the purified compounds against human cancerous cell lines. 

## Conclusion

Two tetrahydrofuran- type sesquiterpenoids (hydroperoxide of davanone (1) and hydroxydavanone (2)) were identified as the active constituents responsible for the cytotoxic property of the petroleum ether extract of *A. aucheri*. Our collective data suggest that hydroperoxide of davanone strongly inhibits cell growth of MCF-7, SK-N-MC, and A2780 cancer cells through induction of apoptosis. Hydroperoxide of davanone induces apoptosis by attenuation of MMP. This process subsequently leads to activate caspase cascade. The findings in this report indicate potential therapeutic value of hydroperoxide of davanone and further research in animal models is necessary to confirm its activity *in-vivo*.
